# Metamortar Composites Reinforced with Re-Entrant Auxetic Cells: Mechanical Performance and Enhanced Energy Absorption

**DOI:** 10.3390/polym17233153

**Published:** 2025-11-27

**Authors:** Jorge Fernández, César Garrido, Luis Muñoz, Felipe Nuñez, Rodrigo Valle, Víctor Tuninetti

**Affiliations:** 1 Department of Mechanical Engineering, Universidad del Bío-Bío, Concepción 4081112, Chile; jfernandez@ubiobio.cl (J.F.); cgarrido@ubiobio.cl (C.G.); 2 Construction Multidisciplinary Research Group, Facultad de Arquitectura, Construcción y Medio Ambiente, Universidad Autónoma de Chile, Talca 3460000, Chile; luis.munoz@uautonoma.cl (L.M.); felipe.nunez@uautonoma.cl (F.N.); 3 Department of Mechanical Engineering, Universidad de La Frontera, Temuco 4811230, Chile

**Keywords:** mechanical characterization, auxetic structures, cellular structures, mortar, energy absorption

## Abstract

This study investigates the mechanical behavior and energy absorption capacity of a novel metamortar composite, developed by embedding re-entrant auxetic cellular structures into a cementitious mortar matrix. Auxetic materials, which exhibit a negative Poisson’s ratio, offer distinct advantages in impact resistance and stress dissipation. Despite their promising properties, their integration into cement-based systems remains limited. In this work, auxetic cells were fabricated using different 3D printing filaments and combined with mortar to form hybrid composites. The specimens were subjected to quasi-static compression tests to evaluate their Young’s modulus, yield strength, and energy absorption capacity. Results indicate that the auxetic inclusions substantially improved the mechanical performance of the mortar, particularly in the case of PLA-based cells, which achieved the highest values across all tested parameters. The enhancements are attributed to the synergistic deformation mechanisms of the auxetic geometry and the surrounding matrix, promoting efficient load distribution and delayed crack propagation. These findings contribute to the advancement of cementitious metamaterials and establish a foundation for scaling toward metaconcrete systems with improved energy dissipation for use in protective, seismic, and infrastructure applications.

## 1. Introduction

In the field of mechanics, composite metamaterials represent a sophisticated integration of microstructural design and diverse material compositions to achieve customized properties. Advancements in industrial technologies have catalyzed ongoing innovations in material development and manufacturing processes [1]. These developments have significantly advanced additive manufacturing (AM), enabling the production of components with complex geometries across multiple scales and material systems. Furthermore, this work builds upon recent modeling advances in the compressive behavior of AM lattice structures [2] and the high-efficiency hysteretic damping capabilities of Kelvin metamaterials [3]. A notable achievement of these innovations is the emergence of microstructured materials, commonly referred to as cellular structures [4]. These materials comprise periodic arrangements of struts and nodes [5], whose geometries can be meticulously engineered to manipulate the macroscopic mechanical behavior [6]. Optimization of such topologies is frequently guided by targeted mechanical performance criteria [7], often drawing inspiration from naturally occurring structures such as wood, bone, or honeycomb patterns [8]. The design of lightweight and functional lattice structures using polymers has become a field of great interest due to their high strength-to-weight ratio and energy absorption capacity, which is essential for applications in structural, biomedical, and aerospace engineering. For example, Ref. [9] illustrates how topological optimization can guide the efficient design of 3D-printed polymer lattices. In the field of structural biomimicry, the review [10] analyzes nature-inspired criteria for optimizing the mechanical performance of these architectures and trace the rapid growth of this research domain through bibliometric analysis [11]. Within this field of research, there is also the analysis of the mechanics of 3D-printed polymer lattices and their dependence on relative density [12]. In Ref. [13], Bustihan et al. explored energy absorption in reusable polymer lattices printed with different flexible polymers, and Wang et al. proposed lattice designs with adjustable anisotropic properties to improve absorption under uniaxial loads [14].

Auxetic materials, characterized by their negative Poisson’s ratio, have attracted growing interest due to their potential to improve mechanical performance and energy absorption in structural and protective systems. These structures, generally based on re-entrant geometries, exhibit lateral expansion under stress, a behavior that allows for greater stiffness and impact resistance. Széles et al. (2024) have recently published the experimental validation of this mechanism. Recent studies confirm that re-entrant and doubly re-entrant auxetic lattices can achieve superior energy absorption and deformation control, outperforming conventional honeycomb architectures [15]. These behaviors arise from the characteristic bending and rotation of inclined struts, which define the plateau stress region during compression [16]. Topology-controlled designs have allowed the mechanical response of auxetic lattices to be adjusted by varying the cell angle, gradient, or slenderness of the strut [17]. Therefore, auxetic structures were selected for their unique ability to combine high stiffness with improved energy absorption, offering a promising strategy for improving the mechanical efficiency of cementitious composites under compressive loading. Recently, it has been shown that the auxetic behavior of re-entrant structures can be tuned by selecting the material and incorporating fillers into the matrix. Tashkinov et al. demonstrated that the addition of polymeric fillers modifies the deformation modes and enhances the auxetic effect in re-entrant structures [18]. Plewa et al. analyzed metallic metamaterials with re-entrant geometry, highlighting their ability to improve strength and damage tolerance under mechanical loads [19]. Furthermore, numerical studies have shown that these configurations exhibit favorable dynamic performance under compression and bending, associated with local bending and rotation mechanisms [20]. These findings reinforce the relevance of re-entrant topologies as strategic candidates for improving energy absorption and ductility in composite systems.

Cementitious composites rank among the most widely utilized construction materials globally. Nevertheless, these materials are inherently characterized by low tensile strength and brittleness, properties that tend to intensify with increasing compressive strength [21,22]. Additionally, factors such as temperature fluctuations, external loads, and other environmental influences can induce internal stresses within cementitious composites, often exceeding their ultimate tensile capacity and leading to crack formation [23]. The propagation of such cracks poses a significant threat to the durability and structural integrity of cementitious materials [24]. Consequently, recent research efforts have concentrated on enhancing the flexibility of these composites and mitigating crack development [25]. To improve the ductility of cementitious composites, various fibers, including natural fibers [26,27], metallic fibers [28,29], and mineral fibers [30,31], have been incorporated into their matrices. However, these fibers exhibit certain limitations, such as poor fiber continuity, challenging processability, low fracture resistance, uneven fiber distribution, and restricted reinforcing efficacy, which collectively hinder advancements in fiber-reinforced cementitious composites [32,33]. Furthermore, during the manufacturing process, fiber agglomeration often occurs, resulting in non-uniform distribution and inconsistent reinforcement. This exacerbates the initiation and propagation of cracks, compromising the overall performance of the composite material [34].

On the other hand, in recent years, researchers have carried out intensive studies on the structural reinforcement of concrete through cellular structures instead of traditional steel and fiber-reinforced cementitious composites [35,36]. Proposing new 3D-printed polymeric structures as reinforcement for cement mortars to improve the performance of cement-based materials [37,38]. The geometric versatility afforded by additive manufacturing facilitates the development of cellular architectures exhibiting mechanical properties that surpass those of conventional materials [39], including elevated strength and stiffness paired with reduced weight [40]. One of the most studied structures whose topology is often inspired by nature are the low-density and high-stiffness honeycomb structures [41]. Cellular structures can be engineered to exhibit superior properties compared to conventional materials, as their mechanical behavior is governed not only by their chemical composition but also by the intricacies of their microscale topology [42,43]. In this way, the mechanical properties of cellular structures can potentially be controlled through topology analysis to meet particular requirements [44]. The existing literature extensively addresses the modeling and experimental characterization of such structures [45,46]. Advances in additive manufacturing technologies have enabled researchers to conduct experimental investigations on these periodic structures, which include both two-dimensional panel configurations and three-dimensional strut-based architectures [47]. One of the cellular structures that has attracted great attention is the auxetic structures. Unlike other structures, these contract laterally when subjected to compression and expand laterally when subjected to tension, which produces a negative Poisson’s ratio [48]. This so-called auxetic effect can generate attractive mechanical properties in structures such as high strength [49], high shear stiffness [50] and high fracture toughness [51]. Some of the most promising auxetic configurations are structures with re-entrant struts, which can exhibit a remarkable energy absorption capacity [52,53], thanks to the fact that the primary deformation of the structure is guided by the bending of its re-entrant struts [54,55]. In this way, a new opportunity is presented to incorporate auxiliary structures as reinforcement in a Mortar matrix. Despite the growing body of work on polymeric and metallic auxetic lattices, the experimental evidence for cementitious composites remains limited. Studies on concrete systems incorporating auxetic reinforcements have been reported, yet data on mortar elements are still scarce [56,57]. The study carried out in [24] demonstrates that the ductility and energy absorption capacity of structural reinforced Mortar samples have been significantly improved and there are mechanical differences depending on the topological configuration of the structure. In Ref. [58], the mechanical behavior of a novel auxetic layered honeycomb Mortar composite was examined under quasi-static compression to assess its performance. The findings indicate that the composite exhibits enhanced strength and ductility, attributed to its higher initial peak stress and stable plateau stress. Furthermore, the auxetic architecture contributes to improved shear strength and overall structural stability. In Ref. [59], the mechanical behavior of an additively manufactured auxetic structure used as reinforcement for cementitious composites was analyzed, aiming to enhance ductility and energy absorption capabilities. The experimental findings reveal that auxetic networks fabricated from ABS polymer exhibit reduced strength and stiffness compared to Mortar, leading to a diminished load-bearing capacity of the reinforced mortar as compressive deformation progresses. Nonetheless, a significant improvement in ductility was observed due to the incorporation of the auxetic networks. The maximum recorded increase in ductility reached approximately 100% and 200%, accompanied by reductions of 15% and 50% in maximum load capacity, respectively.

The incorporation of auxetic panels within a Mortar matrix has been shown in the literature to induce a significant enhancement in the material’s ductility [60,61,62,63]. In this way, the integration of microstructured materials in the design of structural components has encountered significant challenges, primarily due to their elevated cost relative to conventional construction methods and the intricate mechanical phenomena associated with their design and analysis [64]. This necessitates a comprehensive investigation into their mechanical performance. Consequently, the research focuses on reinforced Mortar elements incorporating cellular architectures remains limited [65,66,67,68]. Moreover, existing studies lack an in-depth experimental evaluation of auxetic structures fabricated using various advanced 3D printing filaments. Recent studies have explored three-dimensional auxetic structures based on known planar configurations by introducing new design parameters to induce an asymmetric response [69,70,71]. Furthermore, research has shown that reinforcing an auxetic structure with a polymeric resin matrix significantly improves the energy absorption capacity [72]. However, the impact of a Mortar matrix on the energy absorption mechanical functionality of these auxetic structures remains largely unexamined. It should be noted that, although periodic auxetic lattices are often necessary to obtain effective metamaterial behavior at the structural scale, this study intentionally employs a single re-entrant auxetic unit cell to isolate and analyze the local mechanisms of reinforcement and energy dissipation transferred to the cementitious matrix. The present findings provide the fundamental understanding necessary before scaling the system to multicellular configurations.

In this study, Young’s modulus, yield strength, and energy absorption were selected as the main mechanical parameters, as they characterize the fundamental aspects of the expected response of the composite system. Auxetic structures made from polymer filaments inherently have a lower elastic modulus than cementitious materials; however, their main functional advantage lies in their ability to withstand large deformations and dissipate energy through the mechanism of negative Poisson’s ratio [19,27,73]. Therefore, by incorporating the auxetic unit cell into the mortar matrix, the objective was not to increase the stiffness of the material, but to improve its ductility and energy absorption capacity, accepting a controlled reduction in stiffness. The evaluation of these three parameters allows for a comprehensive assessment of the stiffness–ductility relationship, the onset of plastic deformation, and the overall energy dissipation capacity induced by the auxetic reinforcement. This theoretical framework underpins the analysis presented in the following sections.

The following sections of this document are organized as follows: Section 2 details the implementation of the auxetic design, the 3D printing filaments used, the dosage used for the manufacture of the Mortar, and testing procedures employed throughout the investigation. Section 3 presents the experimental findings and analyzes the mechanical performance in relation to the auxetic mechanisms and material properties described. Finally, Section 4 summarizes the key conclusions drawn from this research and explores potential gaps for future investigation.

## 2. Materials and Methods

### 2.1. Auxetic Structures Manufactured with an FDM System

**Figure 1:**
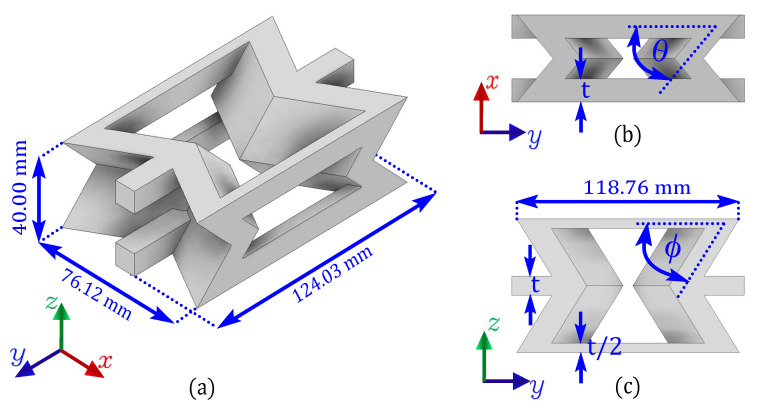
(**a**) Unitary cell of the re-entrant auxetic structure, showing its characteristic geometric parameters: (**b**) re-entrant angle θ, (**c**) internal cell angle ϕ and wall thickness *t*.

In this research, an auxetic cell designed to act as an energy absorbing core is used, based on the graded configuration presented in [74]. However, a constant re-entrant angle will be considered throughout the thickness direction of the cell, as shown in Figure 1. Although the gradation of the angle enhances the bending modulus, this research will focus on improving the mechanical strength and energy absorption capacity of the Mortar structure. In this way, based on previous studies [69,70,71,72] on the design and mechanical characterization of auxetic structures with re-entrant struts, it is known that the smaller the re-entrant angle the structure may exhibit increased ductility. Similarly, when the re-entrant angle is larger, the structure becomes stiffer under compression. Therefore, in this work, the structure is designed with an angle $\theta =45^{\circ }$ to increase the ability to experience energy absorption and an angle $\phi =60^{\circ }$ to increase the stiffness. Furthermore, the thickness of the walls of the structure is t=10.67 mm.

In this way, this unit cell will be used to reinforce a Mortar block with the aim of improving its energy absorption capacity. Therefore, the auxetic unit cells were manufactured using five different commercial filaments for 3D printing and selected to evaluate the influence of polymer type and short fiber reinforcement on the mechanical response of the composite. The filaments used were as follows: (i) Creality brand Polylactic Acid (PLA) filament, a thermoplastic commonly used due to its dimensional stability and ease of printing; (ii) ABS-SP40 and ABS-FRO, both Acrylonitrile Butadiene Styrene copolymers, with ABS-FRO containing flame-retardant additives (UL94 V-0 rating); (iii) PA-GF, a polyamide reinforced with approximately 15% short glass fibers by weight; (iv) PA-CF15, a polyamide reinforced with approximately 15% short carbon fibers by weight. The fiber-reinforced filaments (PA-GF and PA-CF15) offer greater rigidity and improved load transfer capacity due to the presence of uniformly dispersed discontinuous fibers. The selection of these five specific filaments was designed to cover a broad spectrum of mechanical properties, ranging from high-stiffness reinforced polymers (PA-GF, PA-CF) to more ductile thermoplastics (PLA, ABS). Since the auxetic mechanism relies on the rotation and bending of the re-entrant ligaments, the intrinsic stiffness and elongation capacity of the base material are critical variables. Testing this variety allowed us to decouple the geometric auxetic effect from the material properties and identify which polymer class best complements the brittle nature of the mortar matrix. Both ABS and polyamide filaments are from Silver 3D. The composition and mechanical properties of all filaments were confirmed using the manufacturer’s technical data sheets. In addition, two variants of Acrylonitrile Butadiene Styrene (ABS), and polyamide (PA) filaments from the Silver 3D brand are used: ABS-SP40, ABS-FRO, PA-GF and PA-CF15. ABS-SP40 is a modified polymer with significantly improved inter-layer adhesion, while ABS-FRO is a UL94 V-0 flame-resistant material. PA-GF is a fiberglass-reinforced material and PA-CF is carbon-fiber-reinforced. Each of the filaments used was 1.75 mm in diameter and was printed on a Creality K1C CARBON system through a 0.4 mm diameter nozzle. All auxetic unit cells were manufactured using FDM with the same printing parameters to ensure uniformity between samples. The print speed was set at 80 mm/seg, with a layer height of 0.20 mm, a fill density of 100%, an extrusion temperature according to the manufacturer’s specifications for each filament, and a bidirectional layer orientation. These constant parameters ensured that differences in mechanical performance were primarily attributed to material characteristics rather than manufacturing variability. All specimens were printed in the xz plane so that the re-entrant ligaments were oriented parallel to the compression loading direction, minimizing inter-layer delamination effects and ensuring that the evaluated mechanical response primarily reflects the intrinsic auxetic deformation mechanisms rather than print-induced anisotropy. The printing parameters for each material are detailed in Table 1.

**Figure 2:**
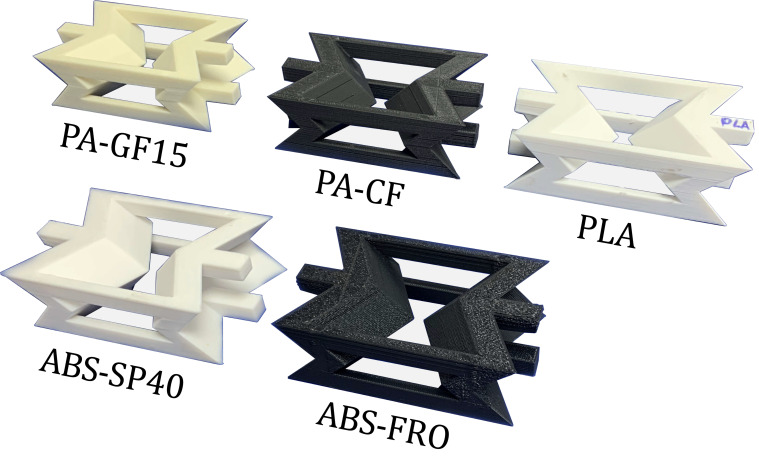
Auxetic structures fabricated with 3D printing using PLA, ABS-SP40, ABS-FRO, PA-GF and PA-CF15 filaments.

According to the printing parameters, unit cells were manufactured for each filament, as shown in Figure 2. Six samples of each structural type were manufactured to ensure statistical reliability, with three auxetic structures and three composite structures per type subjected to quasi-static compression. This setup enables the calculation of an average response during experimentation.

**Figure 3:**
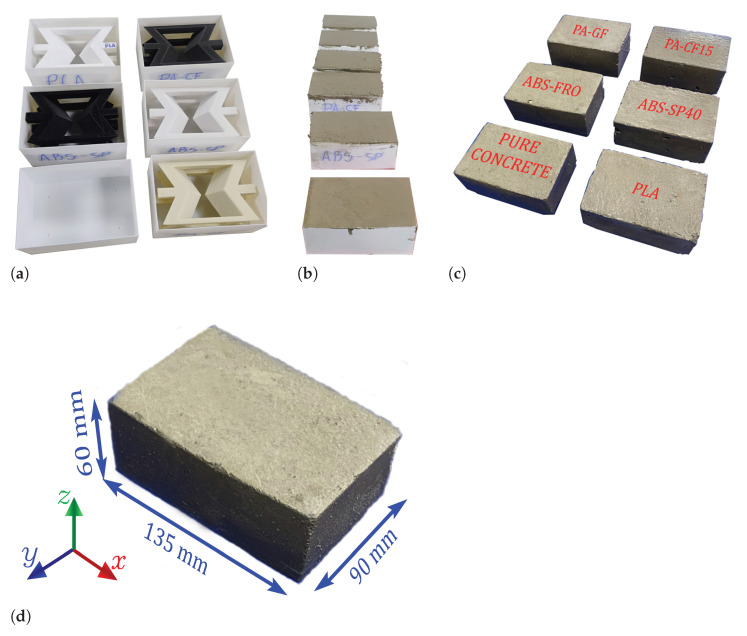
Manufacturing process of additively fabricated auxetic structures (**a**), which were reinforced with a Mortar matrix (**b**) to obtain new composite blocks (**c**), with dimensions of 90 mm × 135 mm × 60 mm (**d**).

Subsequently, three samples of each type of structure were used as reinforcement within a Mortar matrix. For fabrication, molds were additively manufactured using PLA filaments with dimensions of 90 mm × 135 mm × 60 mm. Each auxetic structure was secured within the mold to ensure a Mortar cover of 10 mm on all faces, as shown in Figure 3. Then, a batch of Mortar was prepared to fill each structure. Additionally, three pure Mortar samples were produced for comparative purposes. Once the composite structures were fabricated, they were placed in a curing pool for seven days before demolding.

The Mortar was developed in a Water/Cement/Sand ratio equal to 1:2:6. This Mortar was mixed according to the provisions of NCh 2256 Of. 2013 and NCh 163 Of. 2013. The cement, sand, and water were mixed until a plastic and homogeneous sample was obtained. With this mixture, the composite blocks were manufactured by filling and compacting them using a vibrating table. Finally, the composite blocks underwent a curing process for seven days, according to the provisions of the NCh 170 Of. 2016 standard.

### 2.2. Experimental Testing

**Figure 4:**
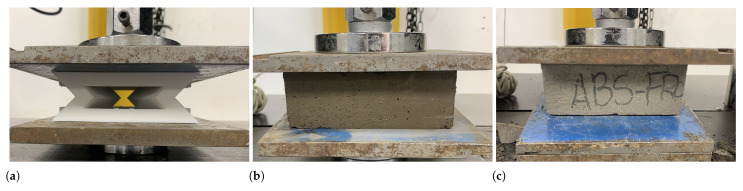
Engineering experimental stresses and strains for each sample: (**a**) additively manufactured auxetic structure, (**b**) Mortar block, and (**c**) composite block.

Once the experimental samples were built, quasi-static compression experiments were carried out to evaluate the energy absorption levels of each sample. All experiments were performed on a Laryee universal testing machine model NE-34100 with a 100 kN load cell and a test speed range ranging from 0.0005 mm/min up to 300 mm/min. For the compression experiments, ASTM*D*-695 was taken as a reference, which establishes the standard test method for compression properties of rigid plastics. In this way, a deformation speed of 1 mm/min was used for the entire range of the test, both in the elastic zone and in the plastic zone. To obtain the experimental results, the machine automatically records the force applied to the sample vs. the displacement of the compression plate. The experimental setup for the compression tests for the auxetic structure, plain mortar, and composite blocks is shown in Figure 4.

### 2.3. Specific Energy Absorption

The scientific literature indicates that auxetic structures characterized by a negative Poisson’s ratio exhibit significant potential to enhance energy absorption capacity under plastic deformation [75,76]. As demonstrated in previous research [72], the integration of an auxetic structure within a homogeneous matrix significantly improves the plastic deformation capacity of the material. This improvement facilitates superior energy absorption, spanning the transition from the termination of elastic behavior to the onset of densification. This transitional phase is referred to as the plateau region of energy absorption. Within this plateau region, the applied force or stress remains approximately constant or nearly unchanged, while the deformation progressively increases. This behavior enables the structure to dissipate maximum energy efficiently without inducing a significant increase in force or stress. Therefore, the plateau region constitutes a critical segment of the response curve, ensuring effective and safe energy dissipation under dynamic loading conditions. The estimation of engineering stress is derived from the energy per unit volume, as represented in Equation (1).

(1) $$\sigma_{p}=\frac{W_{p}}{\Delta_{e}}=\int_{\varepsilon_{y}}^{\varepsilon_{d}}\sigma \left(\varepsilon \right)d\varepsilon$$

The parameter $\sigma_{p}$ denotes the area enclosed under the stress–strain curve within the range defined by $\varepsilon_{y}$ and $\varepsilon_{d}$. In addition, to evaluate and compare the energy absorption efficiency of auxetic structures with that of their Mortar-reinforced counterparts, specific energy absorption (SEA) will be determined. This calculation incorporates the density ρ of each specimen, as defined by the following equation:

(2) $$SEA=\frac{1}{\rho }\times \int_{\varepsilon_{y}}^{\varepsilon_{d}}\sigma \left(\varepsilon \right)d\varepsilon$$

In the subsequent section, the experimental results corresponding to auxetic structures fabricated using various filament materials are detailed. The materials used include PLA, ABS-SP40, ABS-FRO, PA-GF, and PA-CF15. Furthermore, the mechanical behavior of these structures is evaluated when incorporated as reinforcement within a Mortar matrix.

## 3. Results and Discussion

**Figure 5:**
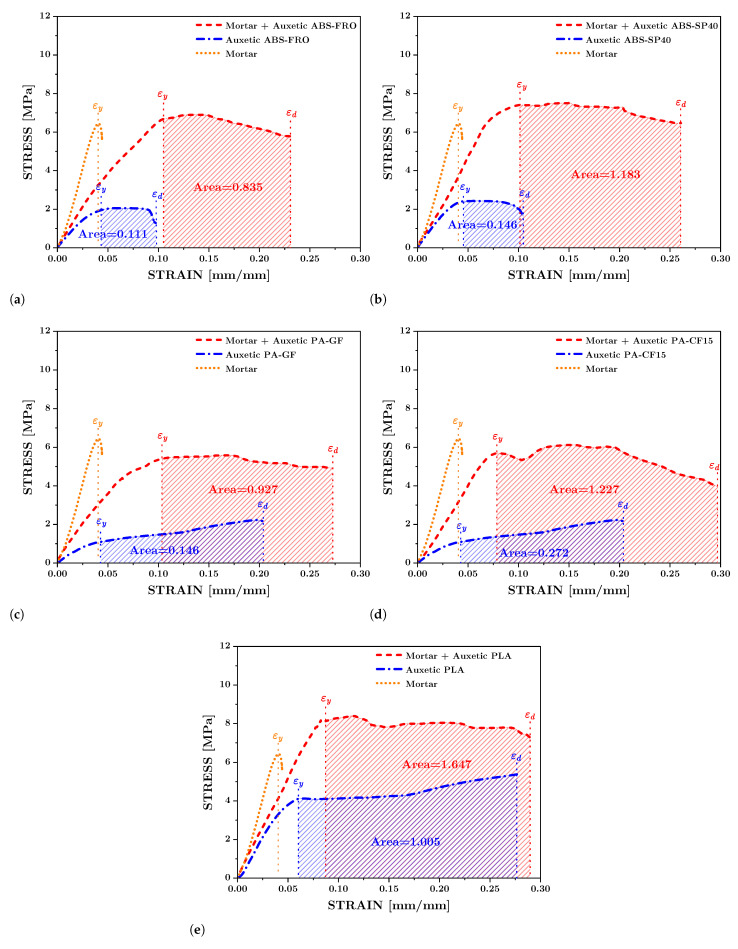
Experimental engineering stresses and strains for each auxetic structure reinforced with Mortar, manufactured with filaments of (**a**) ABS-FRO, (**b**) ABS-SP40, (**c**) PA-GF, (**d**) PA-CF, and (**e**) PLA.

**Figure 6:**
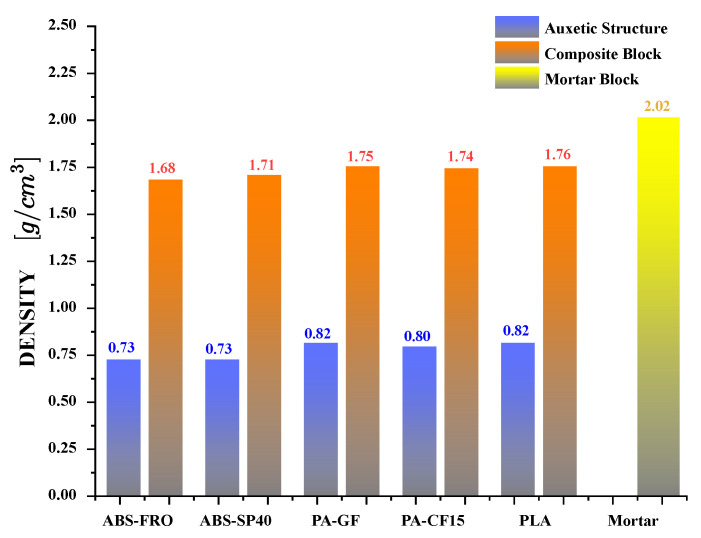
Measured density for each experimental sample under quasi-static compression.

**Figure 7:**
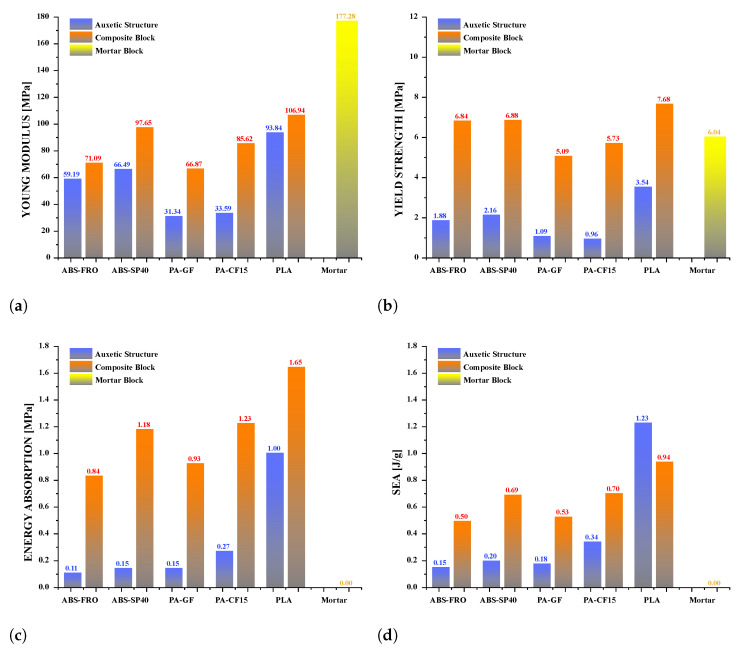
Analysis of experimental results under compression, in which the mechanical behavior of each auxetic structure is compared with composite blocks and Mortar. (**a**) Young’s modulus, (**b**) yield strength, (**c**) energy absorption capacity, and (**d**) specific energy absorption (SEA) are analyzed.

To study the mechanical behavior of reinforced Mortar with the auxetic structure and evaluate how its energy absorption capacity changes, 33 different experiments were carried out. Three auxetic structures were used for each filament type: PLA, ABS-SP40, ABS-FRO, PA-GF, and PA-CF15. Likewise, three auxetic structures made of each type of filament were used to reinforce Mortar blocks. In addition, three pure Mortar blocks were used to evaluate how their mechanical performance changes when reinforcement is incorporated. The experimental results for each composite structure agree well with computer simulations, which allows comparisons to be made to estimate mechanical properties. The experimental values are shown in Figure 5, the measured density of each experimental sample is shown in Figure 6, while the analysis of mechanical performance is shown in Figure 7.

In general, it is observed that the auxetic structure can undergo plastic deformation 17%, while pure Mortar exhibits no plasticity. However, when the auxetic structure is combined with the Mortar matrix, the resulting composite block can achieve plastic deformations of approximately 27%. Thus, by integrating the auxetic reinforcement within the Mortar matrix, this novel composite material demonstrates enhanced mechanical behavior, unattainable by its individual constituents alone. Consequently, this composite enables the development of a new block with improved energy absorption capacity. Figure 5 presents the experimental stress vs. strain curves of the reinforced Mortar blocks with the five distinct additively manufactured structures, where this mechanical behavior is evident. On the one hand, it is possible to observe the variations in Young’s Modulus and Yield Strength of the Mortar structure when incorporating the auxetic structure as reinforcement. As shown in Figure 5a, the pure mortar block has the highest Young’s modulus, while the auxetic structures have a significantly reduced modulus (average decrease of ∼68%). In contrast, the composite blocks exhibit an intermediate modulus, approximately 51% lower than that of pure mortar. The pure Mortar block demonstrates the highest Young’s modulus, while the auxetic structures present a significantly reduced Young’s modulus, with an average decrease of approximately 68%. In contrast, the composite blocks show an intermediate Young’s modulus, which is 51% lower than that of pure Mortar. Specifically, for the reinforced composite block with the structure PLA, Young’s modulus exhibits a reduction of only 39% relative to that of the pure Mortar block, as shown in Figure 7a. However, naturally additively manufactured auxetic structures produced with plastic filaments exhibit a yield strength significantly lower than that of Mortar blocks. However, in most cases, the yield strength of composite blocks closely resembles that of the Mortar block, with a difference of approximately 11%. However, this similarity does not apply to composite blocks incorporating PLA structures, as these exhibit a substantially higher Yield Strength than the unreinforced Mortar block, achieving an increase of up to 27%. Similarly, for the composite blocks reinforced with auxetic structures fabricated from ABS-FRO and ABS-SP40, these demonstrate a yield strength that is marginally superior to that of Mortar, with an approximate increase of 12%, as shown in Figure 7b. Similar to the research conducted in [72], the results indicate the development of a novel reinforced Mortar block with an auxetic structure, exhibiting enhanced mechanical strength and substantially improved ductility compared to conventional Mortar, which typically displays brittle behavior.

It is evident that auxetic inclusion consistently improves ductility in composite blocks, as demonstrated by greater rupture deformations and a more distributed cracking pattern compared to plain mortar (Figure 5a,b). However, the effect on mechanical strength depends on the inclusion material: PLA-reinforced composites showed a notable increase in yield strength (∼+27% compared to mortar), ABS-based composites showed a moderate increase (∼+12%), while PA-GF- and PA-CF15-reinforced composites showed small reductions in yield strength (∼−16% and −5%, respectively). These results indicate that, although auxetic geometry promotes energy dissipation and ductility in all tested configurations, the overall strength response depends on the stiffness and adhesion characteristics of the filament material interacting with the mortar matrix. It is evident that the auxetic structure not only enhances ductility but also contributes to an increase in the mechanical strength of the Mortar. In composite blocks reinforced with auxetic structures fabricated from PA-GF and PA-CF15, a slight reduction in yield strength of 16% and 5%, respectively, is observed. However, in these cases, the capacity to endure plastic deformations increases significantly, allowing for plastic deformation tolerances of 27% and 30%, respectively. On the other hand, to compare the mechanical behavior of additively manufactured auxetic structures with that of composite blocks and pure Mortar blocks, the densities of each sample are integrated into the analysis. The measured density for each experimental sample is presented in Figure 6. The auxetic structures fabricated with different filaments exhibit a comparable apparent density, approximately 0.78 g/cm^3^. Similarly, the composite blocks demonstrate an apparent density of approximately 1.73 g/cm^3^. Consequently, incorporating the auxetic structure as reinforcement within the Mortar block results in a density reduction of approximately 14%. Although the incorporation of the auxetic structure reduces the total density of the block, the mechanical behavior of the composite is strongly influenced by the interaction at the interface between the polymer structure and the mortar matrix. In this system, load transfer is mainly governed by mechanical interlocking and friction restriction, given that the polymer–cement bond is relatively weak. This interfacial confinement stabilizes ligament rotation, delays crack localization, and contributes to the observed increase in deformability and energy absorption. This is significant as it demonstrates the accomplishment of a novel composite structure that exhibits enhanced mechanical strength while maintaining a considerably lower density relative to Mortar. The synergistic deformation mechanism is driven by the kinematic incompatibility between the matrix and the reinforcement. Under compressive load, the cementitious matrix exhibits a positive Poisson’s ratio (lateral expansion), whereas the re-entrant auxetic core exhibits a negative Poisson’s ratio (lateral contraction). This interaction creates a confinement zone at the interface: the mortar physically restricts the rotation of the auxetic ligaments, stabilizing the structure, while the contracting auxetic core creates inward traction that counteracts the tensile stresses responsible for vertical splitting in the mortar. This bidirectional restriction explains why the composite exhibits higher ductility and energy absorption than the sum of its individual components. Simultaneously, the mortar restricts the deformation of the auxetic ligaments, reducing localized instability and delaying the appearance of cracks. This bidirectional restriction generates a more distributed deformation field and contributes to greater energy absorption compared to either component alone. This result demonstrates the feasibility of integrating auxetic structures as reinforcement within a Mortar matrix to enhance its mechanical strength and, most notably, its ductility. Such an approach could enable applications in the development of Mortar blocks optimized for lightweight structural construction. On the other hand, the detachment of the cementitious matrix shows that the interfacial adhesion between the 3D printing polymers and the cementitious matrix is relatively weak, which can reduce the efficiency of load transfer. Future work will explore strategies to improve compatibility between these 3D printing polymers and the cementitious matrix, including increasing surface roughness to enhance mechanical interlocking, chemical or plasma surface activation to improve wettability, and the application of silane- or mineral-based coupling primers to promote chemical bonding at the interface. These approaches have the potential to further improve the strength, toughness, and overall integrity of the composite.

As observed in Table 2, the mortar block exhibited a brittle failure mode, characterized by rapid structural collapse and the formation of multiple vertical cracks along its height. This behavior is consistent with the brittle nature of unreinforced cementitious materials. However, composite blocks reinforced with auxetic structures showed a more ductile response, evidenced by smaller and less frequent cracks propagating in diagonal directions. This crack pattern suggests an effective stress distribution mechanism provided by the auxetic cellular structure, which helps delay crack initiation and reduce their propagation rate. In particular, samples reinforced with polyamides PA-GF and PA-CF experienced mild damage. Future research aims to study crack propagation in reinforced blocks using Digital Image Correlation (DIC). In addition, Figure 7c,d present the energy absorption capacity and the analysis of this property as a function of the density of each structure, in accordance with Equations (1) and (2), respectively. From this, it can be observed that Mortar on its own exhibits negligible energy absorption capacity. However, this brittle behavior undergoes a substantial transformation when an auxetic structure is integrated as reinforcement. The auxetic structure fabricated with *PLA* filaments exhibits the highest energy absorption capacity, averaging approximately 1.26 MPa. It should be noted that the absolute energy absorption values remain relatively low compared to high-tenacity engineered materials. However, the relevance of the result lies in the relative improvement over the pure reference mortar, with an increase of approximately 78% observed in the PLA-reinforced composite. This indicates that the auxetic inclusion promotes a more distributed deformation process and delays the localization of cracks within the mortar matrix. Similarly, the composite block reinforced with the PLA-based auxetic structure achieves an exceptional energy absorption level of 1.56 MPa. Nevertheless, composite structures reinforced with ABS-SP and PA-CF filaments achieve significant energy absorption levels, reaching values of 1.21 MPa and 1.26 MPa, respectively.

Finally, by considering the density of each experimental sample, the specific energy absorption (SEA) can be calculated, as illustrated in Figure 7d. In this way, it is observed that the composite block reinforced with the auxetic PLA structure achieves an SEA of 0.89 J/g, outperforming the blocks reinforced with ABS-SP and PA-CF structures by 20% and 18%, respectively. However, the auxetic structure fabricated with PLA demonstrates an impressive SEA of 0.96 J/g, even exceeding the performance of its Mortar reinforced counterpart by 7%. This demonstrates that this type of cellular structure could serve as an innovative construction system for the development of lightweight structures with high mechanical strength.

## 4. Conclusions

This study investigated the mechanical performance of metamortar composites reinforced with 3D-printed re-entrant auxetic structures. Based on the quasi-static compression results, the following conclusions are drawn:

- The inclusion of auxetic structures significantly enhanced the energy dissipation capacity of the mortar. The most effective reinforcement was the PLA-based structure, which achieved an energy absorption of 1.56 MPa, representing a 78% increase compared to plain mortar.
- While plain mortar exhibited brittle failure at 4.4% strain, the auxetic composites demonstrated superior ductility, achieving failure strains between 23% and 30%. This confirms that the auxetic core effectively delays catastrophic failure.
- As expected, the introduction of the polymeric core reduced the Young’s modulus of the composite by 39% to 62% compared to pure mortar. However, the yield strength was largely maintained, with PLA composites showing a 27% increase in yield strength.
- Among the five filaments tested, PLA provided the optimal balance between stiffness and ductility for this specific application, outperforming the carbon-fiber- and glass-fiber-reinforced polyamides (PA-CF, PA-GF), which proved too stiff to fully exploit the auxetic deformation mechanism before matrix failure.

Future work will focus on optimizing the interfacial bonding between the polymer and cement paste to further enhance the synergistic load transfer.

## Figures and Tables

**Table 1:** Summary of printing parameter settings on Creality K1C CARBON system.

Filament	Extrusion Temp. [°C]	Bed Temp. [°C]	Refrigeration [%]
PLA	220	60	100
ABS-SP40	235	100	0
ABS-FRO	235	100	0
PA-GF	250	100	0
PA-CF15	250	100	0

**Table 2:** Maximum strain and crack propagation behavior observed in mortar and composite blocks under loading.

Sample		Maximum Strain	
	Mortar	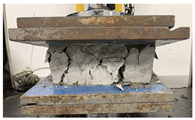	ε=4.4%
COMPOSITE BLOCKS	PLA	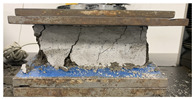	ε=29%
	ABS-FRO	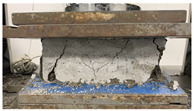	ε=23%
	ABS-SP40	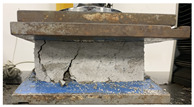	ε=26%
	PA-GF	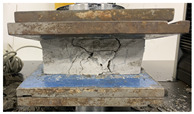	ε=27%
	PA-CF15	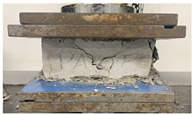	ε=30%

## Data Availability

The original contributions presented in this study are included in the article. Further inquiries can be directed to the corresponding authors.
